# Electrochemical Sensor Based on Iron(II) Phthalocyanine and Gold Nanoparticles for Nitrite Detection in Meat Products

**DOI:** 10.3390/s22155780

**Published:** 2022-08-02

**Authors:** Svetlana I. Dorovskikh, Darya D. Klyamer, Anastasiya D. Fedorenko, Natalia B. Morozova, Tamara V. Basova

**Affiliations:** Nikolaev Institute of Inorganic Chemistry SB RAS, 3 Lavrentiev Pr., 630090 Novosibirsk, Russia; dorov@niic.nsc.ru (S.I.D.); klyamer@niic.nsc.ru (D.D.K.); fedorenko@niic.nsc.ru (A.D.F.); mor@niic.nsc.ru (N.B.M.)

**Keywords:** iron phthalocyanine, gold nanoparticles, nitrite detection, amperometric response

## Abstract

Nitrites are widely used in the food industry, particularly for the preservation of meat products. Controlling the nitrate content in food is an important task to ensure people’s health is not at risk; therefore, the search for, and research of, new materials that will modify the electrodes in the electrochemical sensors that detect and control the nitrate content in food products is an urgent task. In this paper, we describe the electrochemical behavior of a glass carbon electrode (GCE), modified with a Fe(II) tetra-tert-butyl phthalocyanine film (FePc(tBu)_4_/GCE), and decorated with gold nanoparticles (Au/FePc(tBu)_4_/GCE); this electrode was deposited using gas-phase methods. The composition and morphology of such electrodes were examined using spectroscopy and electron microscopy methods, whereas the main electrochemical characteristics were determined using cyclic voltammetry (CV) and amperometry (CA) methods in the linear ranges of CV 0.25–2.5 mM, CA 2–120 μM in 0.1 M phosphate buffer (pH = 6.8). The results showed that the modification of bare GCEs, with a Au/FePc(tBu)_4_ heterostructure, provided a high surface-to-volume ratio, thus ensuring its high sensitivity to nitrite ions of 0.46 μAμM^−1^. The sensor based on the Au/FePc(tBu)_4_/GCE has a low limit of nitrite detection at 0.35 μM, good repeatability, and stability. The interference study showed that the proposed Au/FePc(tBu)_4_/GCE exhibited a selective response in the presence of interfering anions, and the analytical capability of the sensor was demonstrated by determining nitrite ions in real samples of meat products.

## 1. Introduction

Sodium nitrite has a number of useful properties, one of which makes this substance indispensable to the food industry. This property provides sausages, and other meat products, with a pink color, which is associated with fresh, high-quality meat. In food products, sodium nitrite is used as a preservative and an antibacterial agent. It prevents the growth of the causative agent of botulism, the metabolic products of which cause severe food poisoning. Nitrites also contribute to the formation of a specific taste and aroma in meat and fish products. At the same time, this dietary supplement is also a poison, an overdose of which may cause severe health consequences. Sodium nitrite is a toxic substance for all mammals; this is because at high concentrations, it binds to hemoglobin in the blood, which causes oxygen starvation in the body as a whole, and in particular, the brain [1]. In addition, when heated (for example, when frying), nitrosamines with carcinogenic properties are formed [2]. The lethal level of nitrites declared by the WHO is in the range of 8.7–28.3 microns [3]; therefore, it is important to develop a fast, cheap, and accurate method for the quantitative determination of nitrite concentrations, especially for controlling the quality of food. In the literature, various early analytical methods were used to determine nitrite concentrations, among them spectrophotometry, chromatography, chemiluminescence, fluorogenic sensing, electrochemical sensing, and so on [4,5,6].

Of these methods, electrochemical sensors are one of the most effective; this is due to their simple operational technique, rapid response, and lack of interference from nitrate ions and other interfering analytes [7]. Electrochemical methods based on the detection of ions formed during the reduction of nitrites are not used in practice, because they suffer from poor sensitivity and are subject to several interferences [8]. The electrochemical oxidation of nitrite ions is preferable, and is based on the direct reaction of its oxidation to nitrate [9]. It should be noted that nitrite oxidation is associated with a relatively higher overpotential when using a bare glass carbon electrode; therefore, the search for, and study of, materials that will modify electrodes that contribute to decreasing overpotential during nitrite oxidation is an urgent task. In addition, the modification of electrode surfaces may provide a way to extend the dynamic range in analytical determinations.

To determine nitrites, various materials are used to modify the surface of electrodes. Among them are conductive polymers, such as PEDOT and polyaniline, in addition to their composites, which have electroactive molecules and metal nanoparticles (MNPs); for example, PEDOT/PAS (PAS is a polyacenic semiconductor) [10], polyaniline/MoS_2_ [11], nano-sized hydroxyapatite/PEDOT [12], and AuNC/PEDOT [13]. Nanocarbon materials and their composites are also widely used in the modification of the electrodes in the electrochemical sensors that detect nitrite ions; for example, rGO/MWCNTs [14], rGO/ferrocene [15], PtNPs loaded Ni(OH)_2_/multi-walled carbon nanotube composites [16], chitosan/Prussian blue nanoparticles in a mixture of graphene nanosheets and carbon nanospheres [17], and many others.

Metal phthalocyanines (MPc) and porphyrins, especially Co(II)Pc and Fe(II)Pc derivatives, are also used for the electrochemical detection of nitrites [18,19,20,21,22] due to their remarkable physicochemical and electronic properties, as well as their excellent catalytic activity and electron mediator capabilities in various electrochemical reactions [23,24,25]. Catalytic activity is mainly observed in phthalocyanine complexes containing electrochemically active metals such as Cu, Co, Fe, and Mn. To modify electrodes, phthalocyanines are usually deposited by spin coating, drop casting [7,20,26], electropolymerization [27], layer-by-layer deposition [28], and through use of the covalent modification approach [29]. Films of unsubstituted MPcs and their fluoro-, chloro-, and tert-butyl substituted derivatives can also be prepared using physical vapor deposition [30,31]; however, attempts to apply them to the modification of electrodes in electrochemical sensors have not been so fruitful.

Noble metal nanoparticles (MNP) by themselves, and in combination with other materials, have also been utilized in a number of electrochemical applications, such as electrocatalysis, electrochemical analysis, and electrochemical sensing. They are used because of their catalytic properties, which usually occur due to chemisorption on the surface. Another important feature of metal nanoparticles is their high surface area to volume ratio, which is important for sensor applications. A synergetic combination of the properties of phthalocyanines and metal nanoparticles is known to result in the improvement of the sensor properties of MNP/MPc hybrid materials [32], including their sensor response to nitrites [33,34,35,36]. Facilitating mediated electron transfer through the use of AuNPs, in combination with phthalocyanine, may also be important in the creation of cellular biosensors [37,38,39]. In most studies, gold nanoparticles were obtained using “wet” methods or electrodeposition. Gas-phase methods, such as physical vapor deposition and chemical vapor deposition, are used less frequently in the modification of electrodes with gold nanoparticles, although they allow nanoparticles to be obtained with a metal content close to 100%, without any surfactants or stabilizers [40].

In this paper, we describe the electrochemical behavior of a glass carbon electrode (GCE) modified with a Fe(II) tetra-tert-butyl phthalocyanine film (FePc(tBu)_4_/GCE), decorated with gold nanoparticles (Au/FePc(tBu)_4_/GCE), which was deposited using gas-phase methods. The composition and morphology of such electrodes were examined using spectroscopy and electron microscopy methods, whereas the main electrochemical characteristics were determined using cyclic voltammetry (CV) and amperometry (CA) methods in 0.1 M phosphate buffer (pH = 6.8). Results showed that the modification of the FePc(tBu)_4_/GCE, using gold nanoparticles, caused the sensor to become more sensitive nitrite ions, and the detection limit substantially decreased. The interference study showed that the proposed Au/FePc(tBu)_4_/GCE exhibited a selective response in the presence of interfering anions, and the analytical capability of the sensor was demonstrated by determining nitrite ions in real samples of meat products. All components of the proposed sensors are comparatively cheap and commercially available. Moreover, they were deposited on the surface of GCE using a physical vapor deposition technique, which makes it possible to control the ratio of components in the heterostructure and to obtain a large number of electrodes in one deposition cycle.

## 2. Materials and Methods

### 2.1. Materials

FePc(tBu)_4_ was prepared by heating a mixture of 4-tert-butylphthalonitrile (Sigma-Aldrich, St. Louis, MI, USA, CAS 32703-80-3) and iron(II) chloride (Sigma-Aldrich, Saint Louis, USA, CAS 7758-94-3) at 220 °C, which was purified by sublimation in a vacuum (10^−5^ Torr). The metallic gold—Au (99.99%)—was provided by the Novosibirsk Refining Company, Ltd., Russia. The sodium nitrite—NaNO_2_ (>99%)—was obtained from Reactive Ltd., Moscow, Russia. The phosphate buffer solution (PBS, 0.1 M), with a pH value of 6.8, was provided by VWR Chemicals LLC, Wayne, PA, USA. The hexaammineruthenium(II) chloride—Ru(NH_3_)_6_Cl_2_ (>99.9%, CAS No. 15305-72-3)—was provided by Sigma Aldrich, Saint Louis, USA. The glassy carbon electrodes—GCE (SIGRADUR^®^ G GGE plates 10 × 10 mm^2^, d = 1 mm)—were provided by Chemservice Ltd., Moscow, Russia. Other reagents (NaCl, NaNO_3_, glucose, sodium glutamate, sodium ascorbate, buffer electrolytes with pH values of 1.7 (phthalate buffer), 3.6 (acetic buffer), 5.6 (ammonium acetate 0.2 M—Sodium citrate tribasic buffer), 7.4 (PBS buffer), 9.2 (sodium tetraborate)) were of analytical grade (Dia-m Ltd., Novosibirsk, Russia) and used as received without further purification.

### 2.2. Electrodes Preparation

Initially, the GCE plates were washed with sulfuric acid and nitric acid, then degreased with sodium hydroxide and ethanol, and finally dried at room temperature. Layers of FePc(tBu)_4_ were deposited by a PVD method on the surface of GCE plates (samples FePc(tBu)_4_/GCE). The deposition conditions were as follows: the total pressure was 5 × 10^−5^ Torr, the evaporation temperature was 450 °C, the deposition time was 1 h, and the substrate temperature was 60 °C. The samples of the Au/FePc(tBu)_4_/GCEs were prepared using PVD of gold onto FePc(tBu)_4_/GCE at P = 10^−6^ Torr. The deposition conditions were as follows: the gold load was 6 mg, the evaporator temperature was 1532 °C, and the substrate temperature was 100 °C.

### 2.3. Instrumentation

The gold content in the Au/FePc(tBu)_4_/GCE sample was determined using inductively coupled plasma atomic emission spectroscopy (ICP-AES), using a high-resolution spectrometer iCAP 6500 Duo (Thermo Fisher Scientific, Waltham, MA, USA). The HCl (ACS reagent, 37%), HNO_3_ (70%, purified by redistillation–99.999% trace metals basis), deionized water (purified with the Direct-Q3 system (Millipore, Burlington, MA, USA) >18 MU/cm), Ar gas (99.999%), and the Gold Standard for ICP TraceCERT^®^, 1000 mg/L Au in hydrochloric acid, were used as reagents to determine the gold content. The sample was washed off with a minimum amount of concentrated HCl and HNO_3_ (3:1), and the solution was injected into plasma using a SeaSpray type nebulizer with a peristaltic pump. The working parameters of the ICP-AES system are as follows: power supply—1150 W; nebulizer argon flow rate—0.70 L min^−1^; auxiliary—0.50 L min^−1^; and cooling—12 L min^−1^. The data acquisition and processing were carried out with the iTEVA (Thermo Scientific, Philadelphia, PA, USA) software.

The chemical state of GCE, FePc(tBu)_4_/GCE, and Au/FePc(tBu)_4_/GCE was investigated using X-ray photoelectron spectroscopy (XPS). The XPS measurements were performed using the FlexPS system (Specs GmbH, Berlin, Germany) with a PHOIBOS 150 analyzer and monochromatic Al Kα radiation at 1486.71 eV. The XPS spectra were fitted using a Gaussian–Lorentzian convolution function after a Shirley background subtraction. The binding energies were calibrated to the C 1 s peak for C–C group at 284.6 eV.

The surface morphology of the GCE, FePc(tBu)_4_/GCE, and Au/FePc(tBu)_4_/GCE was investigated using a scanning electron microscope (JEOL–JSM 6700 F, Tokyo, Japan) and atomic force microscope (Solver Pro, Moscow, Russia).

The cyclic voltammetry (CV) and amperometry measurements were carried out using a potentiostat–galvanostat (P8-S, Electrochemical Instruments Ltd., Moscow, Russia) to evaluate the electrochemical activity of the studied electrodes in relation to the NO_2_^−^ ions, and to determine their sensory characteristics in relation to NO_2_^−^. The electrochemical cell E-7SF (Electrochemical Instruments Ltd., Moscow, Russia) was assembled using a conventional three-electrode system: a saturated Ag/AgCl reference electrode, a Pt wire auxiliary electrode, and the prepared working electrodes (GCE, FePc(tBu)_4_/GCE, or Au/FePc(tBu)_4_/GCE). All potentials referred to this reference electrode.

CV curves of the GCE and FePc(tBu)_4_/GCE were recorded in the range of potentials from −300 to 1200 mV. In the case of the Au/FePc(tBu)_4_/GCE, along with peaks of nitrite ion oxidation, gold oxidation peaks (at 1060 mV) were also recorded in this region (Figure S1, Supporting Information (SI)). Since the process of gold oxidation at potentials above 1060 mV proceeds irreversibly, the CV curves of the Au/FePc(tBu)_4_/GCE were recorded in the potential range of −300–1000 mV in order to avoid degradation of the samples. The concentration of NO_2_^−^ varied from 0.25 to 2.5 mM. The sweep rates (*v*) ranged from 100 to 500 mV/s.

The CV curves of the FePc(tBu)_4_/GCE and Au/FePc(tBu)_4_/GCE samples were recorded at a nitrite concentration of 1.25 mM, and at a scanning rate of 100 mV/s, with a change in the pH of the electrolyte in the range of 1.7 to 9.2.

The active surface areas of the GCE, FePc(tBu)_4_/GCE, and Au/FePc(tBu)_4_/GCE were determined using a Ru^2+^/Ru^3+^ redox system, in accordance with the Randles–Sevcik Equation (1) for reversible systems [36]:

*I*_pa_ = 2.69 × 10^5^*n*^3/2^*ACD*^1/2^*υ*^1/2^, (1)

where *D* and *C* are the diffusion coefficient (7.7 × 10^−6^ cm^2^/s [41]) and concentration of the redox probe (2 × 10^−6^ mol/cm^3^ [Ru(NH_3_)_6_]Cl_2_), respectively; *n* is the number of electrons transferred (*n* = 1), υ is the scan rate (0.001 V/s), and *A* is the active surface area of the electrode.

The surface coverage of the electroactive sites of the modified GCE samples was determined from the corresponding CV curves (See Section 3.2) using Equation (2) [36]:

*Г* = *Q*/*nFA*, (2)

where *Г* is the surface coverage, *Q* is determined by integrating the area under the anodic peak, *n* is the number of transferred electrons (*n* = 1), *F* is the Faraday constant, and *A* is the active surface area of the electrode.

The amperometric studies of FePc(tBu)_4_/GCE and Au/FePc(tBu)_4_/GCE were performed at *E* = 1 V in an Ar (99.999%, Company “Chistye Gasy”, Novosibirsk, Russia) atmosphere. To test the selectivity of the Au/FePc(tBu)_4_/GCE, NaCl, NaNO_3_, glucose, sodium glutamate, and sodium ascorbate (Na(AA)) were used as interfering compounds. The amperometric measurements were carried out in 0.1 M PBS (pH 6.8) at 1.0 V vs. Ag/AgCl. The concentration of each interfering compound (except Na(AA)) was 50 μM, which is 25 times higher than the concentration of NO_2_^−^ (2 μM). The concentration of Na(AA) was 20 μM, taking into account the data concerning the standard Na(AA)/NaNO_2_ ratios in real meat products, as described in the literature [42,43]. The stability of the response of the Au/FePc(tBu)_4_/GCE to nitrite was studied using amperometric measurements at 24, 48, and 168 h. The reproducibility of the Au/FePc(tBu)_4_/GCE was investigated by CV, measuring five independently prepared identical sensors.

### 2.4. Preparation of Meat Food Samples

Samples of meat products (smoke sausage, sausage produced by the Siberian Food Company Ltd., Novosibirsk, Russia) were purchased at a local supermarket (Supermarket “Yarche”, Novosibirsk, Russia). To study the electrochemical characteristics, the food samples were pretreated, in accordance with a method described elsewhere [7,18]. In brief, preparation of the supernatant solution involved the following steps: 5.0 g of the food samples were cut into small pieces and mixed with 20 mL of distilled water. The mixture was transferred into a grinder, and it was squeezed until the mixture was crushed. This was followed by ultrasonication for 30 min. After the ultrasonic treatment, the supernatant solution was filtered and heated at 70 °C for 40 min to remove traces of proteins and to make the supernatant solutions homogeneous. Finally, the supernatant solution was diluted in 100 mL of 0.1 M PBS solution (pH 6.8) for further electrochemical analysis of nitrites. To prevent the degradation of nitrites, the supernatant solution was stored at −10 °C.

### 2.5. Statistical Analysis

Based on the recommendations given in [6], the main validation parameters (precision, repeatability, and trueness) for the nitrite determination method, using the amperometric calibration curve for Au/FePc(tBu)_4_/GCE (See Section 3.8), were assessed. A standard addition method was used for calibration in the laboratory.

The accuracy parameter was assessed by calculating the relative standard deviation (RSD) for five parallel determinations, using standard solutions, with a certain concentration of nitrite. To assess repeatability, the analysis of three replicates of 5 μM standard solutions was performed on the same day; a week later, it gave a 5% RSD value.

The trueness was determined through the recovery evaluation. Five parallel detections, using standard solutions with a certain concentration of nitrite, were carried out in order to determine the recovery of the proposed calibration; this was defined in terms of the ratio of found NO_2_^−^ concentrations to the added concentration: %R = 100(C_found_)/C_added_).

## 3. Results and Discussion

### 3.1. Characterization of the FePc(tBu)_4_/GCE and Au/FePc(tBu)_4_/GCE

**Figure 1:**
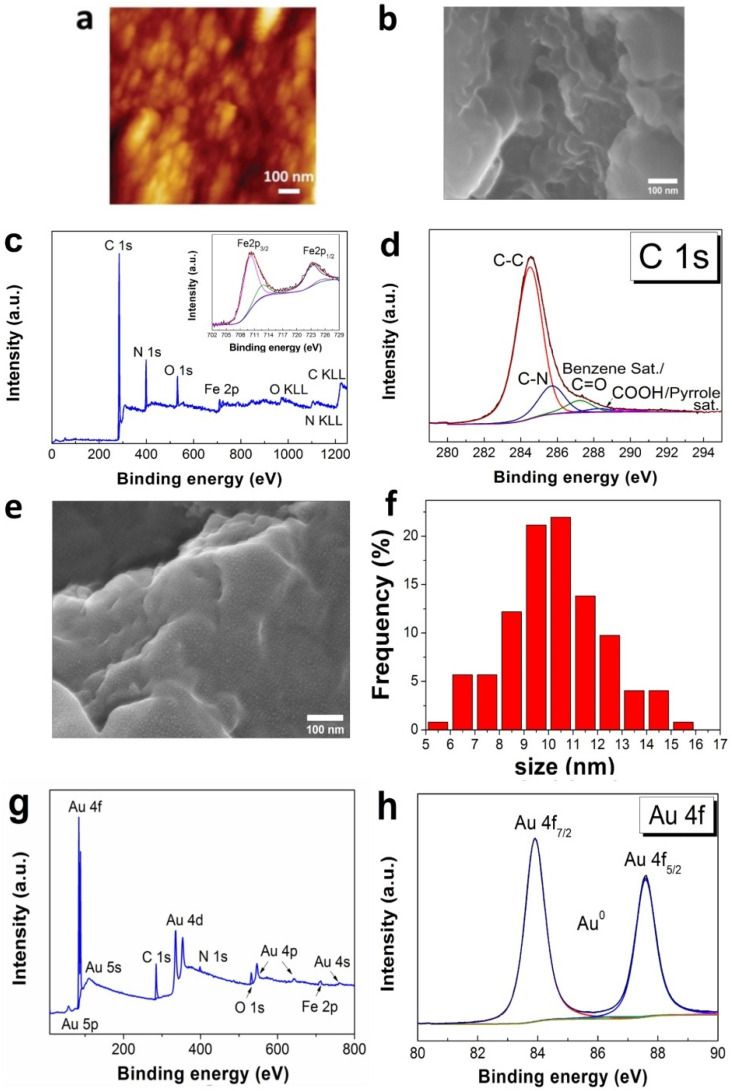
AFM image of a bare GCE (**a**); SEM image of the FePc(tBu)_4_/GCE (**b**); XPS spectrum of the FePc(tBu)_4_/GCE with Fe 2p (**c**); fitting of the C 1s spectra (**d**); SEM image of the Au/FePc(tBu)_4_/GCE (**e**) with AuNPs size distribution (**f**); XPS spectrum of the Au/FePc(tBu)_4_/GCE (**g**); and fitting of the Au 4f spectra (**h**).

The surface of a bare GCE is porous, and it is formed by oval agglomerates with dimensions of 80–200 nm (Figure 1a,b). The EDS data are given in Figure S2 (SI). The average roughness of the bare GCE is 112 nm (scan area 10 × 10 μm^2^). The FePc(tBu)_4_ film repeats the topography of the bare GCE. According to XRD analysis, the FePc(tBu)_4_ film is amorphous. In the XPS spectrum of the FePc(tBu)_4_/GCE sample, the characteristic C 1s, N 1s, and Fe 2p lines, which are related to the FePc(tBu)_4_ films, were observed (Figure 1c). The Fe 2p spectra consist of two peaks: one asymmetric peak at around 711 eV, which come from the electrons at the Fe 2p_3/2_ level, and another, less intense peak at approximately 724 eV, which come from Fe 2p_1/2_ electrons. The fitting components at 709.9 and 723.1 eV are assigned to Fe^2+^ [44]. The low intense lines at 712.3 and 725.2 eV may be due to a decrease in electron density around the Fe atom; this is because of the delocalization of the π-electron system during the adsorption of FePc(tBu)_4_ on the GCE matrix [45].

An estimation of the carbon concentration, in relation to the iron phthalocyanine, shows that the C 1s spectrum is mainly determined by the FePc(tBu)_4_ structure. Since FePc(tBu)_4_ has an extended π-system, it is characterized by the presence of ‘shake-up’ satellites in the C 1s and N 1s spectra [46,47,48,49]. Thus, all of the observed C 1s peaks (Figure 1d) at 284.6 eV (C-C), 285.8 eV (C-N), 287.3 eV, and 288.4 eV can be a attributed to the FePc(tBu)_4_ film. The second peak, observed at about 285.8 eV, comes from the eight pyrrole carbon atoms of FePc(tBu)_4_. The low intense C 1s peak, observed at about 287.3 eV and 288.4 eV, is associated with the benzene and pyrrole carbon shake-up transitions of FePc(tBu)_4_ [47,48,49]; however, the regions of the C 1s peaks, at approximately 284.6 eV, 287.3 eV, and 288.4 eV, are associated with the carbon atoms in the phthalocyanine rings. Moreover, small contributions can be produced by the aromatic carbon, C=O, and COOH groups in the GCE, respectively. In the N 1s spectra of the FePc(tBu)_4_/GCE (Figure S3, Supporting Information), the line at 398.8 eV can be associated with two nonequivalent nitrogen types that cannot be distinguished in the spectrum due to their almost identical binding energies, whereas the low-intensity line at 400.3 eV can be attributed to a satellite structure, which is a ‘shake-up’ of four pyrrolic nitrogens Fe-N [46,47,48]. The O 1s spectra (Figure S3) show two components that are related to a carbon-bound oxygen (531.8 eV) and a small amount of adsorbed water or a OH group (533.5 eV). Quantitative analysis shows that the N: Fe concentration ratio is close to eight, which is within the XPS margin of error.

After the gold deposition, uniformly distributed AuNPs are visualized on the surface of the Au/FePc(tBu)_4_/GCE sample (Figure 1e). The average sizes of AuNPs in Au/FePc(tBu)_4_/GCE are 9–11 nm (Figure 1f). Along with Au 4f lines, the low-intensity Fe 2p, C 1s, and N 1s lines, which are related to FePc(tBu)_4_, are observed on the XPS spectrum of the Au/FePc(tBu)_4_/GCE (Figure 1g), thus confirming the formation of the Au/FePc(tBu)_4_ heterostructure on the GCE surface. The positions of the main Au 4f peaks indicate the presence of metallic gold (Au 4f_7/2_ 84.1 eV) (Figure 1h). From the EDS data (Figure S2), the composition of the Au/FePc(tBu)_4_/GCE (at.%) is C: 54.1; Au: 21.3; N: 16.1; O: 5.6; Fe: 2.3; and Si: 0.6. The average gold content in the Au/FePc(tBu)_4_/GCE, determined using ICP-AES, is 4.7 ± 0.5 μg/cm^2^.

### 3.2. Charge Transfer Behavior of GCE, FePc(tBu)_4_/GCE and Au/FePc(tBu)_4_/GCE

**Figure 2:**
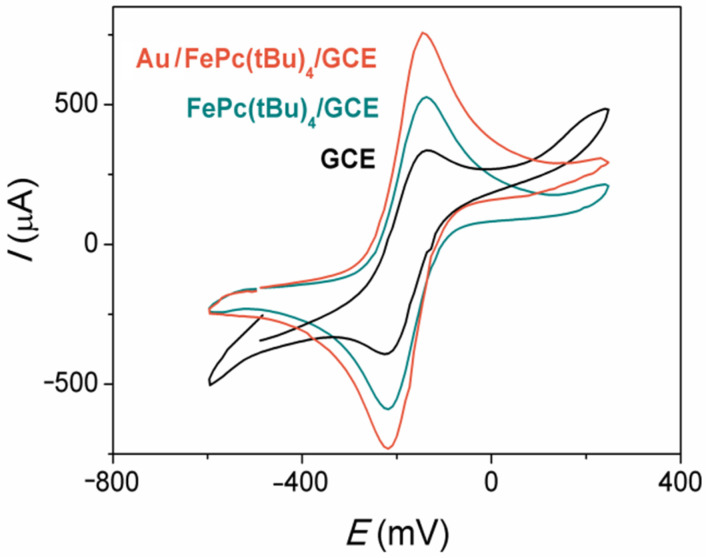
CV curves of the bare GCE, FePc(tBu)_4_/GCE, and Au/FePc(tBu)_4_/GCE in 2 mM [Ru(NH_3_)_6_]Cl_2_ in 0.5 M KCl. Scan rate is 9.9 mV/s.

The charge transfer behavior of the GCE, FePc(tBu)_4_/GCE, and Au/FePc(tBu)_4_/GCE samples were studied using CV measurements in a solution containing 2 mM [Ru(NH_3_)_6_]Cl_2_ (Figure 2). Hexaammineruthenium (II)/(III) is a universal and convenient couple that is used to study the electron transfer behavior of an electrode, as well as to estimate the electrode surface area [50,51]. Recent works emphasized the advantages of using this couple due to its stability during electron transfers, and the absence of the co-called ‘aerial oxidation’ effect [52]. This couple is also used to study the electrochemical properties of gold-containing electrodes [53,54].

The bare GCE demonstrates a reversible behavior during the single electron transfer of the Ru^2+^/Ru^3+^ redox reaction, with a peak-to-peak separation (Δ*E*_p_) value of 84 mV (Table 1, column 2). The modification of a GCE surface with FePc(tBu)_4_ film or a Au/FePc(tBu)_4_ heterostructure leads to a decrease in the values of Δ*E*_p_. Both the FePc(tBu)_4_/GCE and Au/FePc(tBu)_4_/GCEs exhibit a reversible behavior in the Ru^2+^/Ru^3+^ redox reaction. A comparison of the current (*I*_pa_) values of the Ru^2+^/Ru^3+^ peak (Table 1, column 3) in the series of GCE, FePc(tBu)_4_/GCE, and Au/FePc(tBu)_4_/GCE samples indicates that the best electron transfer behavior is exhibited in the Au/FePc(tBu)_4_/GCE. It is assumed that the presence of AuNPs provides a high surface-to-volume ratio, thus contributing to good electron transfers between the redox probe and the Au/FePc(tBu)_4_/GCE.

The average active surface area values of the GCE, FePc(tBu)_4_/GCE, and Au/FePc(tBu)_4_/GCE were calculated from three identical samples of each type, and they are given in Table 1. Their average roughness factors (the ratios of *A* values to geometrical surface area) are 1.46, 1.59, and 1.84, respectively. The *Г* values were found to be 0.9 × 10^−9^ and 2.2 × 10^−9^ mol·cm^−2^ for the FePc(tBu)_4_/GCE and Au/FePc(tBu)_4_/GCE, respectively. These values are comparable to, or even higher than, those of other electrodes that are modified with metal phthalocyanines [55] or AuNPs [35,56].

Thus, the modification of a bare GCE with FePc(tBu)_4_ film or a Au/FePc(tBu)_4_ heterostructure provides a high surface-to-volume ratio, which therefore makes these samples promising for further use as electrochemical sensors.

### 3.3. Effect of the Electrolyte pH on Nitrite Oxidation

**Figure 3:**
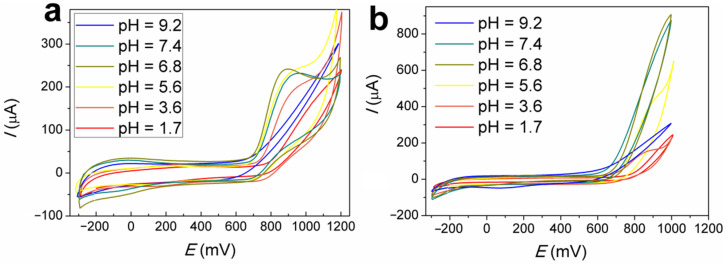
CVs curves of the FePc(tBu)_4_/GCE (**a**) and Au/FePc(tBu)_4_/GCE (**b**), recorded in electrolytes with various pH balances, containing 1.25 mM NaNO_2_, at a scanning rate of 100 mV/s.

The effect of electrolyte pH on the potentials and current values of nitrite oxidation, as it occurs on the FePc(tBu)_4_/GCE and Au/FePc(tBu)_4_/GCE, was studied in order to select the optimal conditions for further investigation of the nitrite oxidation response. FePc(tBu)_4_ demonstrates a reversible behavior during single-electron transfer, in a pH range of 1.7 to 7.4 (Figure 3a). The positions of both of the redox peaks Fe^2+^/Fe^3+^ and the nitrite oxidation peaks shifted towards positive potentials with increasing proton concentrations. It is possible that an excess of anions (pH < 3.5) and hydroxy groups (pH > 7.5) blocked the active centers of the electrode, thus hindering electron transfer [57]. The same trend was almost observed on the FePc(tBu)_4_/GCE.

Nitrite ions are known to have a low stability in acidic media [58], and the efficiency of the nitrite ion oxidation process decreases with an increase in pH > 7.5 [34,59]. Consequently, the highest current values of nitrite oxidation for both electrodes are in the pH range of 5.6–7.4. The maximal nitrite oxidation current, together with the lowest oxidation potential (905 mV), is achieved at a pH value close to six; this caused greatest level of activity on FePc(tBu)_4_/GCE. After decorating the FePc(tBu)_4_/GCE with AuNPs, its proclivity for nitrite oxidation increases, and the current nitrite oxidation on this electrode reaches a maximum of pH = 7; therefore, the electrolyte with a pH = 6.8 is optimal for studying the electrochemical behavior of both the FePc(tBu)_4_/GCE and Au/FePc(tBu)_4_/GCE with regard to nitrite oxidation.

### 3.4. Cyclic Voltammetry Characterization of GCE, FePc(tBu)_4_/GCE, and Au/FePc(tBu)_4_/GCE

**Figure 4:**
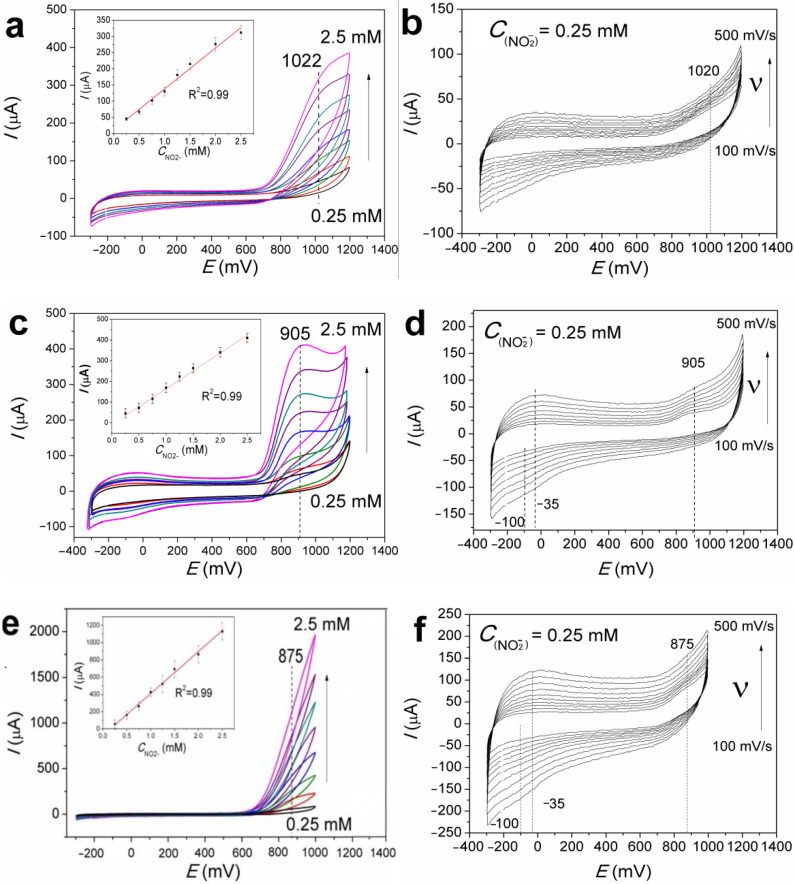
CV curves of the bare GCE, FePc(tBu)_4_/GCE, and Au/FePc(tBu)_4_/GCE in the nitrite concentration range of 0.25 to 2.5 mM (**a**,**c**,**e**) and at different scan rates from 100 to 500 mV/s (**b**,**d**,**f**).

From the CV curves of the GCE (Figure 4a), FePc(tBu)_4_/GCE (Figure 4c), and Au/FePc(tBu)_4_/GCE (Figure 4e), at a recorded pH = 6.8, in the range of nitrite concentrations from 0.25 to 2.5 mM, the peaks associated with the oxidation of nitrate ions (anodic branch) were detected at 1020, 905, and 875 mV, respectively.

The peaks related to the redox processes of FePc(tBu)_4_ are observed at −100 and −35 mV, as in the cases of the FePc(tBu)_4_/GCE and Au/FePc(tBu)_4_/GCE (Figure 4). An increase in the peak current of nitrite oxidation (*I*), on the CV curves of the studied samples, with an increase in the concentration of NO_2_^−^, indicates a high sensitivity to the studied electrodes; this is especially true of the Au/FePc(tBu)_4_/GCE in the oxidation reaction with nitrite ions. It should be noted that in the studied series of samples, peaks related to the nitrate ion reduction process are absent on the CV curves. This indicates that the nitrite irreversibly oxidizes on the surfaces of these electrodes. The shift of the peaks during the oxidation of nitrite ions from 1020 to 875 mV indicates that the modification of the GCE surface facilitates the electrochemical oxidation process on the surfaces of the FePc(tBu)_4_/GCE and Au/FePc(tBu)_4_/GCE.

According to the data in the literature, peaks corresponding to nitrite oxidation lie in the range of 0.8–1 V (PBS, pH = 5.8–7) in the case of electrodes that are modified with transition metal phthalocyanines [21,24,33,34,59], and in the range of 0.6–0.9 V in the case of electrodes that are modified with AuNPs (PBS, pH = 5–8) [34,35,36,60]. The potential values of the FePc(tBu)_4_/GCE and Au/FePc(tBu)_4_/GCE studied in this work correspond well with the data in the literature, which also indicates their possible use in sensors which determine the presence of nitrites.

### 3.5. The Mechanism of Nitrite Oxidation on the FePc(tBu)_4_/GCE and Au/FePc(tBu)_4_/GCE

**Figure 5:**
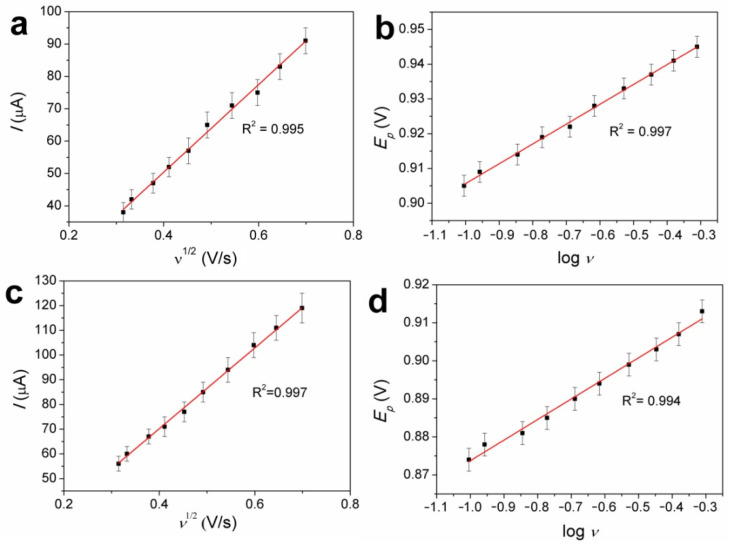
*I*-*υ*^1/2^ and *E*-log*υ* dependences for the FePc(tBu)_4_/GCE (**a**,**b**) and Au/FePc(tBu)_4_/GCE (**c**,**d**).

To gain insight into the mechanism of nitrite oxidation, which occurred on both the FePc(tBu)_4_/GCE and Au/FePc(tBu)_4_/GCE, CV curves at different υ (100–500 mV/s) in 0.25 mM nitrite were recorded (Figure 4b,d,f). For both the FePc(tBu)_4_/GCE and Au/FePc(tBu)_4_/GCE, linear dependencies between the current peaks of nitrite oxidation and the square of the scan rates are shown in Figure 5a,c, and the corresponding dependencies are given in Table 2. The linearity of these dependencies may indicate that the process of nitrite oxidation on the FePc(tBu)_4_/GCE and Au/FePc(tBu)_4_/GCE is controlled by the mass transport of nitrite ions from the bulk solution to the electrode surface.

To determine the number of electrons that participate in the rate-determining step (*n*α) and the electron transfer coefficient (α) values of the FePc(tBu)_4_/GCE and Au/FePc(tBu)_4_/GCE, the Laviron’s expressions concerning the anodic peak potential of NO2^−^ (*Ep*) vs. the logarithm scan rate (logυ) were obtained (Figure 5b,d). The corresponding linear regression equations are listed in Table 2. Applying the Tafel equation (Equation (3)), the (1−α)*n*_α_ values of the FePc(tBu)_4_/GCE and Au/FePc(tBu)_4_/GCE were calculated (Table 2, column 4)

*Ep* = (2.3*RT*/2[(1 − α)n_α_]*F*)log*v* + C, (3)

where *R*, *T*, and *F* values represent the universal gas constant, temperature, and Faraday’s constant, respectively. From the estimated (1 − α)n_α_ values for the FePc(tBu)_4_/GCE and Au/FePc(tBu)_4_/GCE, the one-electron transfer in the rate-determining step (rds) was suggested. The Tafel values were calculated to be 116 and 108 mV/decade for the FePc(tBu)_4_/GCE and Au/FePc(tBu)_4_/GCE, respectively. According to the work of Partra et al. [61], a Tafel slope of less than 120 mV/dec indicates the high efficiency of an electron transfer and the low passivation of the electrode surface. The Au/FePc(tBu)_4_/GCE studied here demonstrates the best electron transfer characteristics; this is due to its increased surface area, which, in turn, is due to the AuNPs. It is important to note that we cannot completely exclude the possible role of the electrocatalytic oxidation of nitrites on Au cores in the range of potentials from −300 to 1000 mV.

In addition, both *I-υ*^1/2^ and *E-logυ* dependences for the Au/FePc(tBu)_4_/GCE (Figure S4) were plotted in the range of −300–1200 mV (Table 2). The Tafel slope for the Au/FePc(tBu)_4_/GCE increases from 108 to 188 mV/decade when the potential range changes. This means that the efficiency of electron transport on the Au/FePc(tBu)_4_/GCE, in the range of −300–1200 mV, decreases because of the passivation of the electrode surface due to subsequent chemical reactions involving gold ions shortly after electron transfer. In this case, it is possible to conclude that the electrocatalytic oxidation of NO_2_^−^ to NO_2_ on Au^0^ nanoparticles likely occurs via adsorption using the inner sphere mechanism [56,62].

The AuNPs present in the modified electrodes form a [Au^0^–NO_2_^−^] complex in the presence of nitrite. This complex is electrochemically oxidized to Au^+^ and NO_2_, regardless of whether the reduction conversion of Au^+^ to Au^0^ is observed at the Au/FePc(tBu)_4_ [56].

Finally, the total number of electrons (*n*) transferred during the oxidation of nitrite ions on the surface of the FePc(tBu)_4_/GCE and Au/FePc(tBu)_4_/GCE (−300–1000 mV) is estimated from the *I*-*υ*^1/2^ dependences (Table 2) using the Randles–Sevcik equation (Equation (4)), thus initiating an irreversible process:

*I* = 2.69 × 10^5^[(1 − α)n_α_]^1/2^*nACD*^1/2^*υ*^1/2^, (4)

where *A* and [(1 − α)n_α_] are values from Table 1 and Table 2, *C* (NO_2_^−^ concentration) is equal to 0.25 × 10^−6^ mol/cm^3^*,* and *D* is equal to 2.1 × 10^−5^ cm^2^/s [63]. From Equation (4), the *n* values are close to two for both the FePc(tBu)_4_/GCE and Au/FePc(tBu)_4_/GCE.

Thus, based on the analysis of the data in the literature [34,64], the following mechanism for nitrite oxidation on the FePc(tBu)_4_/GCE and Au/FePc(tBu)_4_/GCE was proposed. In the first stage, the Fe^II^Pc(tBu)_4_ undergoes oxidation to Fe^III^Pc(tBu)_4_ (Equation (5)), then, the formation of the adduct [Fe^II^Pc(tBu)_4_―NO_2_] takes place (see Equation (6)), whereas Equation (7) demonstrates the one-electron rate-determining step (rds). We believe that the deposition of AuNPs onto the FePc(tBu)_4_/GCE is accompanied by a decrease in the Tafel slope (due to an increase in the efficiency of the electron transfer to the Au/FePc(tBu)_4_/GCE compared with the electron transfer to the FePc(tBu)_4_/GCE). Equation (8) describes the regeneration of the initial [Fe^II^Pc(tBu)_4_] with the formation of NO_2_, which then undergoes a disproportionation reaction (see Equation (9)), thus resulting in the formation of NO_2_^−^ and NO_3_^−^ ions:

[Fe^II^Pc(tBu)_4_] → [Fe^III^Pc(tBu)_4_]^+^ + e (5)

[Fe^III^Pc(tBu)_4_]^+^ + NO_2_^−^→ [Fe^II^Pc(tBu)_4_―NO_2_] (6)

[Fe^II^Pc(tBu)_4_―NO_2_] → [Fe^III^Pc(tBu)_4_―NO_2_]^+^ + e (rds) (7)

[Fe^III^Pc(tBu)_4_―NO_2_]^+^ + 2e→ [Fe^II^Pc(tBu)_4_] + NO_2_ (8)

2NO_2_ + H_2_O → NO_3_^−^ + NO_2_^−^ + 2H^+^ (9)

### 3.6. Nitrite Detection

**Figure 6:**
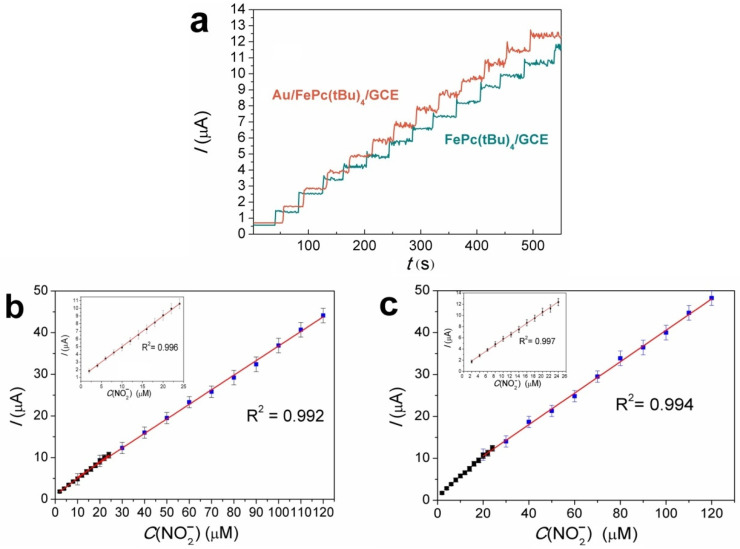
Amperometric response of the FePc(tBu)_4_/GCE and Au/FePc(tBu)_4_/GCE after the addition of nitrite (2–26 μM) (**a**). Calibration plots of the peak current versus the NO_2_^−^ concentration for the FePc(tBu)_4_/GCE (**b**) and Au/FePc(tBu)_4_/GCE (**c**).

Amperometric experiments with FePc(tBu)_4_/GCE and Au/FePc(tBu)_4_/GCE were carried out at 1 V under constant stirring conditions to induce a better interaction between the electrode and the electrolyte. Figure 6a shows a gradual increase in the oxidation current on the *I*-*t* curves of both samples after each addition of 2 μM of NO_2_^−^. As a result of the amperometric experiments on the FePc(tBu)_4_/GCE and Au/FePc(tBu)_4_/GCE electrodes, which were carried out in the NO_2_^−^concentration ranges of 2–26 μM and 20–120 μM, respectively, the calibration curves shown in Figure 6b,c were obtained.

Calibration curves demonstrate two different linear regions for both investigated electrodes. The curves in the concentration range from 2 to 26 μM are described by the following equations: *I*(μA) = 0.675 + 0.362 *C*_NO2_^−^ (R^2^ = 0.996) and *I*(μA) = 0.87 + 0.455 *C*_NO2_^−^ (R^2^ = 0.997) for the FePc(tBu)_4_/GCE and Au/FePc(tBu)_4_/GCE, respectively. The calculated limits of detection (LOD) (S/N = 3) were 0.63 and 0.35 μM for the FePc(tBu)_4_/GCE and Au/FePc(tBu)4/GCE, respectively (Table 3). The sensitivities were 0.36 and 0.46 μAμM^−1^ for the FePc(tBu)_4_/GCE and Au/FePc(tBu)_4_/GCE, respectively, whereas the sensitivity values per unit of the active surface of the electrode (*A*, Table 1) were 0.8 and 0.87 μAμM^−1^ cm^−2^.

The other linear regions, with slightly different slopes, are observed at NO_2_^−^ concentrations ranging from 20 to 120 μM (Figure 6b,c). These linear dependencies are described by the equations *I*(μA) = 1.79 + 0.334 *C*_NO2_^−^ (R^2^ = 0.992) and *I*(μA) = 3.65 + 0.419 *C*_NO2_^−^ (R^2^ = 0.994) for the FePc(tBu)_4_/GCE and Au/FePc(tBu)_4_/GCE, respectively (Figure 6b,c). A comparison of these dependencies indicates a 5–6% decrease in the sensitivity of both electrodes at higher concentrations of nitrite. This effect is probably associated with a decrease in electrochemically active centers due to the formation of a large amount of product on the electrode surface [65].

The results showed that the LOD of the Au/FePc(tBu)_4_/GCE is two times lower than that of the FePc(tBu)_4_/GCE. This is likely to be due to an increase in the surface area of the Au/FePc(tBu)_4_/GCE, due to the deposition of AuNPs. The analytical performance of the investigated sensors is compared with other nitrite sensors in Table 3. The literature analysis shows that the surface modification of GCE or graphene surfaces with AuNPs seems to be a good strategy for improving the characteristics of electrodes; this is due to the excellent conductivity of gold and the formation of active centers.

For example, the sensitivity of a GCE, modified with Au particles that are sized between 100–110 nm, obtained by electrodeposition, followed by 3-mercaptopropionic acid self-assembly, thus enabling attachment of an iron(III) monoamino-phthalocyanine via amide bond formation, was quite low [34]. On the other hand, composite materials with AuNPs particles demonstrated higher sensitivity values, which indicates the advantages of using composite materials for electrode modification due to their synergistic effects [56,65,66]. According to [7], the high sensitivity of the electrodes can be explained by the efficiency of the electron transfer between the modified electrode and nitrite. In our case, the modification of the FePc(tBu)_4_/GCE with small AuNPs has had a positive effect on electron transfer (see Section 3.4), and the Au/FePc(tBu)_4_/GCE exhibits a higher sensitivity compared with the FePc(tBu)_4_/GCE and some of the electrodes shown in Table 3.

**Table 3:** Comparison of the analytical performance of the FePc(tBu)_4_/GCE and Au/FePc(tBu)_4_/GCE with other nitrite sensors.

Electrode	Method (pH)	Concentration Range	LOD (μM)	Sensitivity (μAμM^−1^)	Ref.
AuNP/rGO HMS/GCE ^a^	Amp (7)	0.5 μM–2.8 mM	0.5	-	[67]
Rose-like AuNPs/ MoS_2_ nanoflower/graphene	Amp (4.7)	0.5 μM–5 mM	1	-	[68]
GCE modified with Pd/CoPc nanorods	DPV (6)	0–5 mM	0.1	0.01	[33]
Nano-Au/Ch/GCE ^b^	DPV (7)	0.7–750 μM	0.1	0.35	[66]
Au-Fe(III) nanoparticle/GCE	DPV (7)	0.3–150 μM	0.3	0.13	[69]
FeMAPc-MPA/AuNPs/GCE	DPV (5.8)	1.9 μM–2 mM	0.21	0.015	[34]
AuNPs/GCE			-	0.008	
Dendrimer/AuNPs/GCE ^c^	Amp (5)	10–5000 μM	0.2	-	[56]
CoTM-QOPc/CNP/GCE ^d^	Amp (7)	0.1–350 μM	0.033	-	[21]
Fe(III)P/MWCNTs/GCE ^e^	Amp (4)	1–600 μM 0.6–1.6 mM	0.5	-	[70]
FePc(tBu)_4_/GCE	Amp (6.8)	2–26 μM (20–120 μM)	0.63	0.36 (0.33)	This work
Au/FePc(tBu)_4_/GCE			0.35	0.46 (0.42)	

^a^ HMS—hollow microspheres, CR-GO—chemically reduced graphene dioxide; ^b^ Ch—Choline; ^c^ Dendrimer/AuNPs—gold nanoparticles and a carbosilane-dendrimer possessing peripheral electronically communicated ferrocenyl units (Dend); ^d^ CoTM-QOPc/CNP—Cobalt (II) tetra methyl-quinoline oxy bridged phthalocyanine, CNT—carbon nanotubes, ^e^ Fe(III)P/MWCNTs—chloro [3,7,12,17-tetramethyl-8,13-divinylporphyrin-2,18-dipropanoato (2−)]iron(III)/multi-walled carbon nanotube composite.

In general, the characteristics of the sensors proposed in this work are comparable to the characteristics of sensors based on other materials, even surpassing some of them in terms of sensitivity. It should also be noted that in most of the works mentioned above, more complex methods and more expensive materials were used to modify the surface of the electrodes. With regard to our proposed sensors, all components are commercially available, and gas-phase deposition methods for both phthalocyanine films and gold nanoparticles make it possible to control the thickness of the layers and the ratio of components in the heterostructure. Moreover, it is also possible to obtain a large number of electrodes in one deposition cycle.

### 3.7. Selectivity, Stability, and Reproducibility of Au/FePc(tBu)_4_/GCE

**Figure 7:**
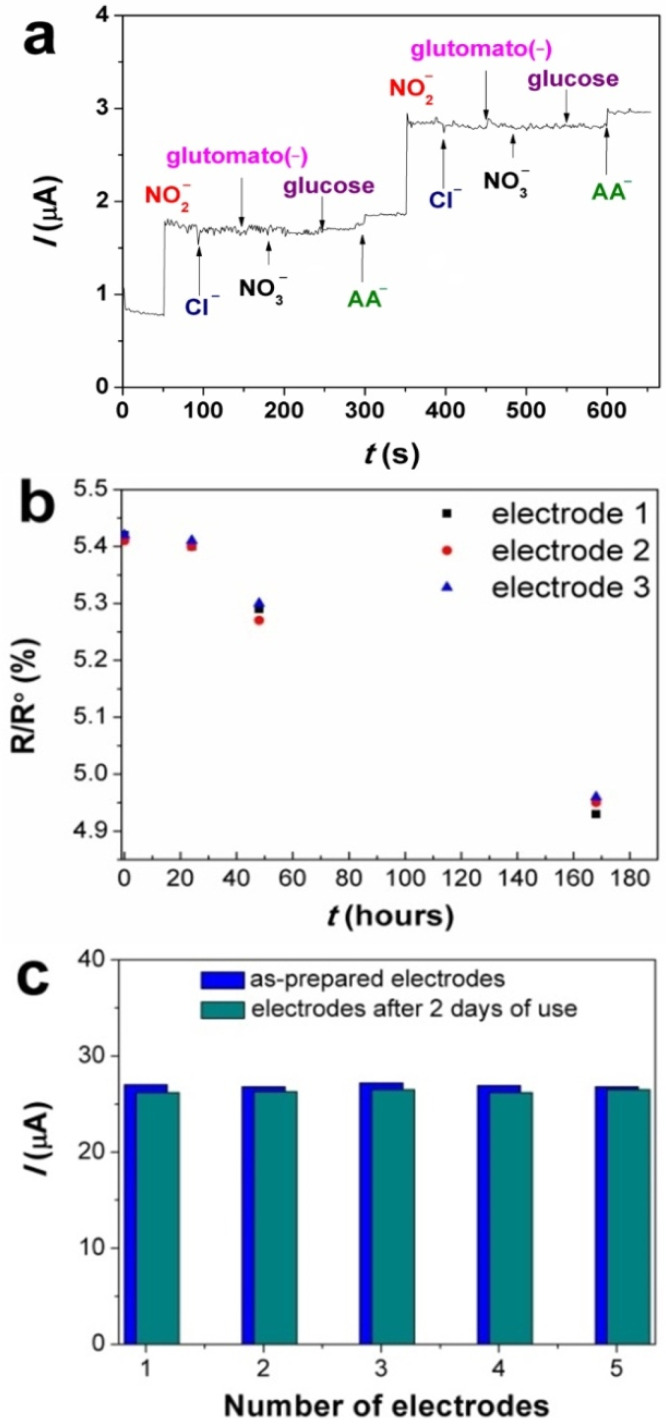
Amperometric response of the Au/FePc(tBu)_4_/GCE to various interfering analytes (**a**); stability of the amperometric response (R) of the Au/FePc(tBu)_4_/GCE after the addition of 10 μM NO_2_^−^ (**b**); and the repeatability and reproducibility of the Au/FePc(tBu)_4_/GCE (**c**).

The Au/FePc(tBu)_4_/GCE, which showed the best LOD and sensitivity in the studied series of samples, was examined in detail to assess its suitability for detecting nitrites in real samples of meat products. For this purpose, interfering analytes, viz. NaCl, NaNO_3_, glucose, sodium glutamate, and sodium ascorbate (Na(AA)), which can be found in real samples of meat products, were selected. Figure 7a shows no obvious change in the current response after sequential injections of interfering ions (signal change less than 3%). Only in the case of *AA*^−^, was a 5.5% signal change observed (from three parallel probes).

The stability response of the Au/FePc(tBu)_4_/GCE to NO_2_^−^ was studied in 0.1 M PBS in the presence of 10 μM NaNO_2_ at a working potential of 1 V. The obtained amperometric current responses of three Au/FePc(tBu)_4_/GCE samples are plotted in Figure 7b. The stability of the Au/FePc(tBu)_4_/GCE response to NO_2_^−^ was studied in 0.1 M PBS in the presence of 10 μM NaNO_2_ at a working potential of 1 V. The obtained amperometric current responses of three Au/FePc(tBu)_4_/GCE samples are shown in Figure 7b. After one day, the sensor response changed by no more than 1%, whereas after two and seven days, it decreased by no more than 2.4 and 5.1%, respectively. Thus, the measured response of the Au/FePc(tBu)_4_/GCE to NO_2_^−^ oxidation shows good stability.

To determine the reproducibility of both, in terms of preparation and reusability, the Au/FePc(tBu)_4_/GCE was evaluated for a series of five identical samples. CV measurements were carried out in 0.1 M PBS with 0.1 mM of NO_2_^−^ under identical experimental conditions (−300−1000 mV, ν = 100 mV/s) (Figure 7c). The relative standard deviation (RSD) values for the anodic peak current were measured using a series of five electrodes. Before and after their operation, the values were estimated to be 1.9% and 2.7%, respectively, which indicates a good reproducibility with regard to the NO_2_^−^ response.

### 3.8. Real Meat Food Sample Analysis Using Au/FePc(tBu)_4_/GCE

The practical applicability of the Au/FePc(tBu)_4_/GCE was verified by determining the presence of NO_2_^−^ in two samples of meat products. In the first stage, preliminary measurements of the concentration of nitrites in both food samples were carried out. Based on these data, the calibration curve *I*(μA) = 0.87 + 0.455 *C*_NO2_^−^ (R^2^ = 0.994) which contained a range of nitrite concentrations, from 2 to 26 mm, was selected. Then, the standard addition method was used to verify the accuracy of the proposed calibration (Table 4, columns 2, 3).

The recovery percentages of nitrite detected in the samples ranged from 97 to 105% (Table 4, column 4), whereas the RSD values did not exceed 7% (Table 4, column 3). The satisfactory recovery and RSD values indicate that the Au/FePc(tBu)_4_/GCE sensor is suitable for detecting nitrites in real samples.

Finally, using this calibration curve, the concentrations of nitrite in both samples were determined and then attributed to the mass of the meat product (5 g, see Section 2.4). The content of nitrites in smoked sausages and sausages (Table 4, column 5) was determined to be 15.56 ± 0.63 and 11.38 ± 0.48 mg/kg, respectively.

A comparison of the nitrite content in the studied samples, with the data published in some reports [7,71,72], showed an adequate concentration of nitrite in both food samples. It should also be noted that the amount of nitrite found in the examined samples of meat products did not exceed the standards recommended by the European Food Safety Authority (EFSA) [73].

## 4. Conclusions

Electrochemical sensors based on a glass carbon electrode (GCE), modified with Fe(II) tetra-tert-butyl phthalocyanine film (FePc(tBu)_4_/GCE) and a heterostructure with gold nanoparticles (Au/FePc(tBu)_4_/GCE), were obtained using a facile gas-phase deposition technique. Their composition and morphology were examined using spectroscopy and microscopy methods. The Au/FePc(tBu)_4_/GCE, with uniformly distributed 9–11 nm AuNPs, exhibited a high surface-to-volume ratio, and its active surface area reached 0.523 nm. All components of the proposed sensors are comparatively cheap and commercially available, and they were deposited on the surface of the GCE via a physical vapor deposition technique; this makes it possible to control the ratio of components in the heterostructure and to obtain a large number of electrodes in one deposition cycle.

The optimal conditions for cyclic voltammetry (CV) experiments involving nitrite oxidation on the FePc(tBu)_4_/GCE and Au/FePc(tBu)_4_/GCE were determined to be as follows. The electrodes should be placed in 0.1 M phosphate buffer (pH = 6.8), in the potential ranges of −300–1200 mV and −300–1000 mV, respectively. The CV results showed that the Au/FePc(tBu)_4_/GCE had the highest efficiency in terms of nitrite oxidation, due to its enhanced current response and the fact that it had the lowest oxidation potential compared with the bare GCE and FePc(tBu)_4_/GCE; this illustrates the potential of combining metal phthalocyanines with gold nanoparticles.

The sensor based on the Au/FePc(tBu)_4_/GCE had a low limit of detection at 0.35 μM, along with a high sensitivity to nitrite ions of 0.46 μAμM^−1^. The proposed Au/FePc(tBu)_4_/GCE demonstrated good repeatability and reproducibility, as well as long-term response stability. The interference study showed that it exhibited a selective response to NO_2_^−^ in the presence of interfering anions, indicating that the proposed Au/FePc(tBu)_4_ heterostructure would be a promising material for the production of sensors that determine nitrite ions in food.

## Figures and Tables

**Table 1:** Δ*E*_p_, *I*_pa_, and A values of the GCE, FePc(tBu)_4_/GCE, and Au/FePc(tBu)_4_/GCE samples.

Electrode ^1^	Δ*E*_p_ (mV)	*I*_pa_ (μA)	*A* (cm^2^)
GCE	84	681	0.413
FePc(tBu)_4_/GCE	80	744	0.451
Au/FePc(tBu)_4_/GCE	76	863	0.523

^1^ Geometrical surface area of the electrode is 0.283 cm^2^.

**Table 2:** *I*-*υ*^1/2^ and E-logυ dependences for the FePc(tBu)_4_/GCE and Au/FePc(tBu)_4_/GCE.

Electrode ^1^	*I*(μA)-*υ*^1/2^(V/s)^1/2^	*E(*V*)*-log*υ*	(1-α)*n*_α_
FePc(tBu)_4_/GCE	*I* = 6.781 + 167.4υ^1/2^ (R^2^ = 0.995)	*E*= 1.343 + 0.058 logυ (R^2^ = 0.997)	0.50
Au/FePc(tBu)_4_/GCE	*I* = 7.833 + 163.9υ^1/2^ (R^2^ = 0.997)	*E*= 1.554 + 0.054 logυ (R^2^ = 0.994)	0.55
Au/FePc(tBu)_4_/GCE	*I* = 8.033 + 190.9υ^1/2^ (R^2^ = 0.998)	*E* = 0.954 + 0.089 logυ (R^2^ = 0.997)	0.31

^1^ Au/FePc(tBu)_4_/GCE was studied at potential range (−300–1200 mV).

**Table 4:** Determination of nitrite in Sample 1 (smoked sausages) and Sample 2 (sausages) using the Au/FePc(tBu)_4_/GCE (*n* = 5).

Food Sample	Added (μM)	Found (μM)	Recovery (%)	Nitrite in Sample mg/kg
Sample 1	3.00	2.91 ± 0.18	97	15.56 ± 0.63
	5.00	5.12 ± 0.27	102	
	9.00	9.29 ± 0.34	103	
Sample 2	3.00	3.15 ± 0.21	105	11.38 ± 0.48
	5.00	4.90 ± 0.29	98	
	9.00	9.24 ± 0.37	103	

## Data Availability

Not applicable.

## Supplementary Materials

The following supporting information can be downloaded at: https://www.mdpi.com/article/10.3390/s22155780/s1, Figure S1: CV curves of Au/FePc(tBu)4/GCE, recorded at (−300–1200 mV); Figure S2: EDX spectrum of Au/FePc(tBu)4/GCE; Figure S3: N 1s spectra of FePc(tBu)4/GCE (a) and O 1s spectra of FePc(tBu)4/GCE (b); Figure S4: *I*-*υ*^1/2^ (a) and *E*-log*υ* (b) dependences for Au/FePc(tBu)_4_/GCE, recorded in the presence of 0.25 mM nitrite at (−300–1200 mV).
