# Supplementary material for: Electrochemical Sensor Based on Iron(II) Phthalocyanine and Gold Nanoparticles for Nitrite Detection in Meat Products
Source: Sensors (Basel). 2022 Aug 2;22(15):5780. doi: 10.3390/s22155780 (PMC9371027; doi:10.3390/s22155780)
Supplement: Supplementary file 1 [file sensors-22-05780-s001.zip › sensors-1817402-supplementary.pdf]

SUPPORTING INFORMATION

Electrochemical Sensor Based on iron(II) Phthalocyanine and Gold Nanoparticles for Nitrite Detection in Meat Products

Svetlana I. Dorovskikh[1], Darya D. Klyamer[1], Anastasiya D. Fedorenko[1], Natalia B. Morozova[1], Tamara V. Basova[1*]

[1] Nikolaev Institute of Inorganic Chemistry SB RAS, 3 Lavrentiev Pr., Novosibirsk, 630090, Russia

[2] Novosibirsk State University, Novosibirsk 630090, Russia

*Correspondence: basova@niic.nsc.ru; Tel.: +73833309556

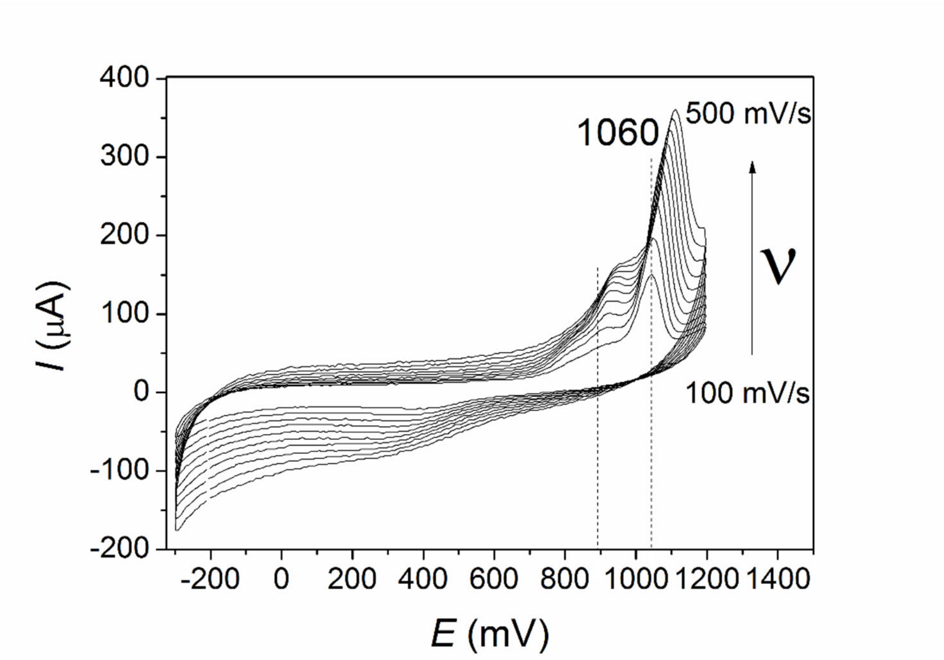

Figure S1. CV curves of Au/FePc(tBu)$_4$/GCE, recorded at (-300–1200 mV).

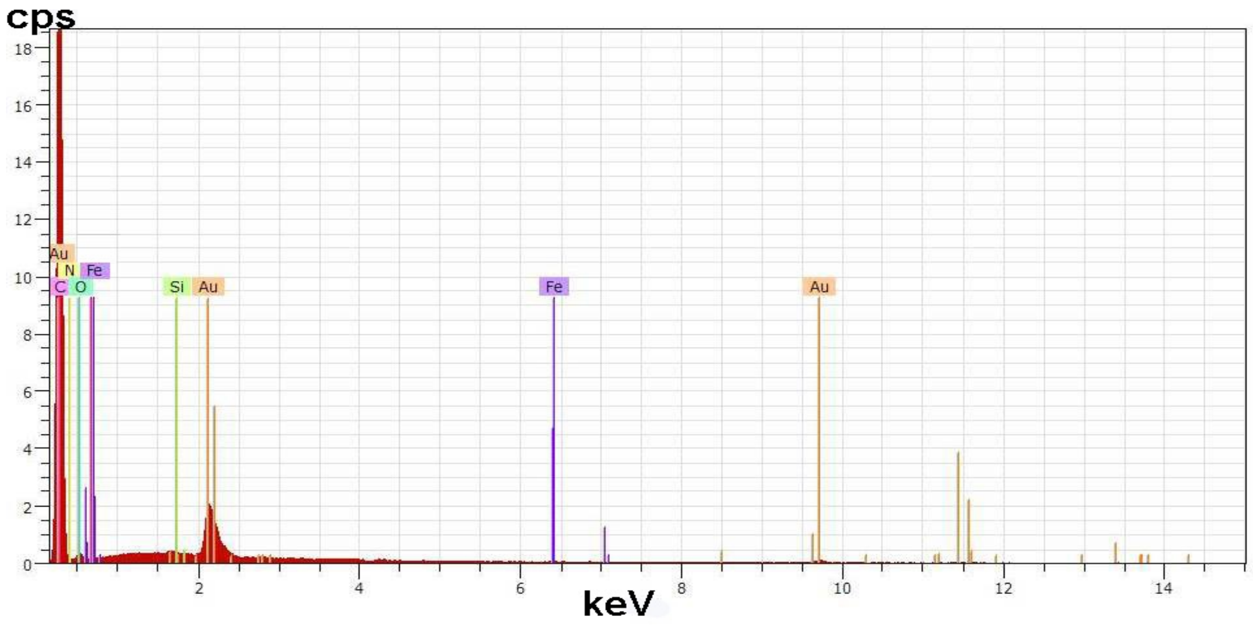

Figure S2. EDX spectrum of Au/FePc(tBu)$_4$/GCE.

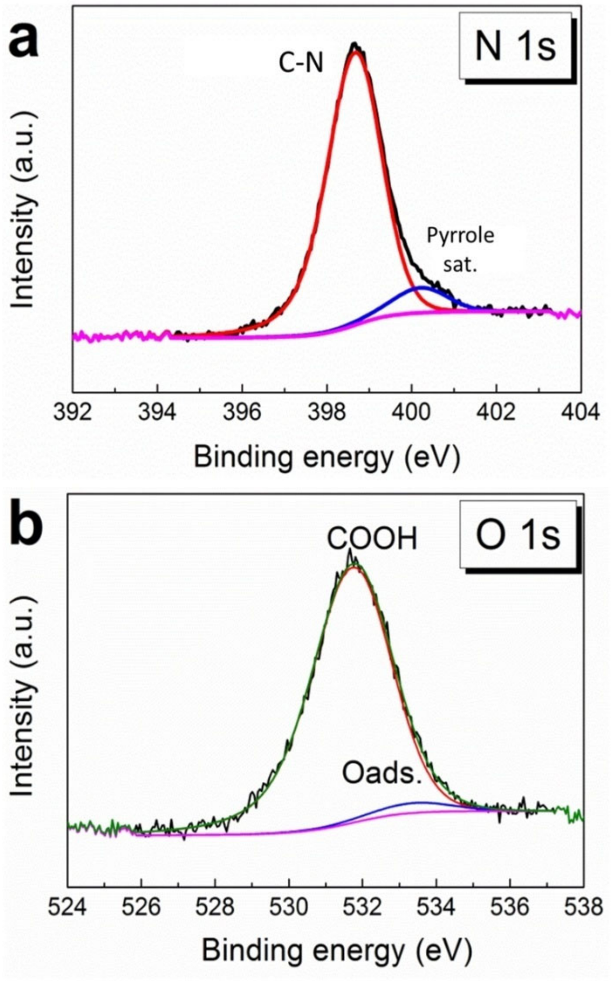

Figure S3. N1s spectra of FePc(tBu)$_4$/GCE (a) and O1s spectra of FePc(tBu)$_4$/GCE (b).

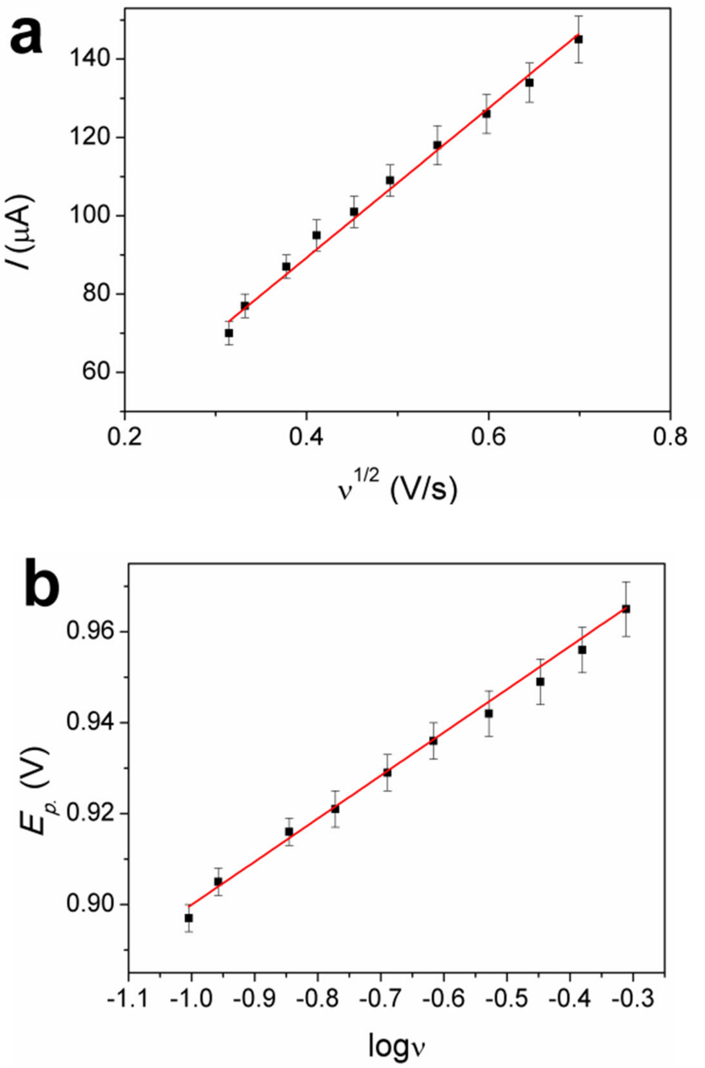

Figure S4. I-$\upsilon^{1/2}$ (a) and E-logυ (b) dependences for Au/FePc(tBu)$_4$/GCE, recorded in the presence of 0.25 mM nitrite at (-300–1200 mV).
